# Report on the Medical Department of the University of Pennsylvania, for the Year 1848: To the Alumni of the School

**Published:** 1848-09

**Authors:** 


*Report on the Medical Department of the University of Pennsylvania, for the year* 1848: *to the Alumni of the School.* By the Medical Faculty.

This report is, in other words, an annual *announcement*, or *circular*, and *catalogue*, of the Medical Department of the University of Pennsylvania for the year 1848. This venerable institution, it will not be forgotten, led the way in extending the term of instruction from four to five months, in partial conformity with the recommendation of the American Medical Association. Of the result of the experiment the report speaks as follows:

"The Faculty have unalloyed satisfaction in stating to the friends of the school, that their expectations in relation to the numbers and character of the class have been greatly exceeded; and they consider the fact to have been established by the experience of the past year, that the improvement in regard to the extension of the courses of instruction in the schools, proposed by the Convention is quite feasible. So far from the apprehended diminution having taken place, the number of students in attendance upon the lectures, as shown by the accompanying catalogue, exceeded that of any former year; and the class of the University for the late session is believed to have been the largest ever assembled upon this continent. The young men came actuated by the spirit of their preceptors, and generally eager for the proposed extension of their study; and most of them remained with an unflagging attention until near the close of the session. It is true that many of those attending upon their first course, left us before the period had expired; but this has always been the case, to a certain extent, even when the length of the session did not exceed four months; and it will be impossible to obviate such a result, until other schools in our neighborhood, as we sincerely hope will ultimately be the case, shall come into the same measure recommended by the Convention."

Some changes have taken place in the Faculty during the last year. Prof. Hare, so long the incumbent of the chair of Chemistry, has resigned, and Prof. James B. Rogers appointed his successor. The chair of Clinical Surgery has been vacated by the disease of Prof. Jacob Randolf, and is now filled by the appointment of Dr. Geo. W. Norris.

The Faculty propose, if the extension of the lecture term to five months be generally adopted by other schools, to extend to six months, so as fully to meet the views of the Association. They add as follows:—

"They wish it to be understood, that they sympathize heartily in the general sentiments of the medical public in relation to the elevation of the profession in character and usefulness; and are prepared to co-operate, so far as the instrumentality of the schools may be required, in all feasible measures for the promotion of that object. The possession of a due amount of primary knowledge by the student is at present, perhaps the point of greatest importance. The Faculty have reason to believe that the classes are yearly improving in this respect; and it is their wish that a gradual advance may take place, until the honors of the Institution shall be considered an evidence as well of general as of professional accomplishment. But the co-operation of the private medical teachers throughout the country is essential; and the Faculty sincerely hope, that increased attention to the very important point of preliminary education, may be among the happy results of the great movement in which the profession is now engaged."

